# 3T CMR for quantification of aortic valve area - A comparison between continuity equation via phase contrast sequences and planimetric analysis

**DOI:** 10.1186/1532-429X-13-S1-P33

**Published:** 2011-02-02

**Authors:** Uwe Speiser, David Haas, Achmed Abbas, Stefanie Jellinghaus, Ruth H Strasser, Steffen Schön

**Affiliations:** 1 Dresden University of Technology, Dresden, Germany

## Introduction

Assessment of aortic valve area (AVA) is primarily carried out by echocardiography in clinical routine. In cases of insufficient acoustic window, discrepant medical findings or suboprimal Doppler conditions, 1.5 T magnetic resonance imaging (MRI) is discussed as an non-invasive alternative imaging method for planimetry of AVA. If quantification of AVA is also possible at higher magnetic field strength had not been investigated until now. The aim of the present study was to quantify AVA using planimetry and continuity equation and correlate them with each other.

## Methods

In 3T CMR phase contrast sequences were performed vertically to blood flow above the aortic valve (AV) and left ventricular outflow tract (LVOT) and the respective flow velocities were determined. Aliasing phenomenon was compensated by gradual increasing of the encoding velocity. Planimetric determination of AVA and LVOT area were executed in SSFP-sequences and magnitude image. The planimetric AVA was correlated with the AVA calculated by continuity equation.

## Results

31 patients with and without aortic valve stenosis were included (43 ± 19 years). Planimetric AVA was 2,6 ± 1,4 cm^2^, the velocities above AV were 235 ± 133 cm/s. The planimetric areas in the LVOT were identified with 115 ±52 cm/s. Calculations of AVA via continuity equation resulted in 2,5 ± 1,5 cm^2^ and correlated to planimetric AVA (r=0,98). The very good correlation between planimetric AVA und AVA determined by continuity equation was verifiable at all AVA category groups. Bland-Altman-analysis did not present any signs of under- and overestimation of both measurement methods.

## Conclusion

At 3T CMR determination of AVA is possible by planimetry as well as by continuity equation. Both methods for quantification of AVA demonstrate a very good correlation without under- or overestimation.

